# Acute unexplained hepatitis in children

**DOI:** 10.2471/BLT.22.020922

**Published:** 2022-09-01

## Abstract

Investigation of an outbreak of acute hepatitis of unknown cause in children may prompt research into other unexplained cases. Vijay Shankar Balakrishnan reports.

Dr Aymin Delgado, a paediatric gastroenterologist at the Joe DiMaggio Children's Hospital in Florida, United States of America (USA), started to notice the first unusual cases of acute hepatitis in children in her clinical practice in mid-2020.

First, there was the 12-year-old girl who was brought into the emergency department with acute coronavirus disease 2019 (COVID-19) and Epstein-Barr virus coinfection.

“The girl had no prior history of liver disease but presented with symptoms that included very elevated liver enzymes (often an indication of inflammation or liver damage), fever and diarrhoea,” Delgado recalls.

Another case came in five months later; this time, a 14-year-old girl who was brought into the emergency department with nausea and vomiting. She too had elevated liver enzyme test results and tested positive for COVID-19.

By July 2022, Delgado had seen eight children with a similar presentation and suspected they were part of an emerging outbreak that includes multiple cases of acute hepatitis of unknown cause in children reported from dozens of countries worldwide, some of which have progressed to liver failure and death.

The initial reports of the disease came from Scotland in the United Kingdom of Great Britain and Northern Ireland in early April. Soon after, the United Kingdom Health Security Agency (UKHSA) reported many more cases in children under the age of 10 years across the United Kingdom.

“There were 44 children who had hepatitis of indeterminate cause and acute liver failure in the first quarter of 2022, up from six in the first quarter of 2021,” says Dr Deirdre Kelly, Professor of Paediatric Hepatology at the University of Birmingham, United Kingdom, and head of the Liver Unit at the Birmingham Women’s and Children’s Hospital.

The reports were quickly followed by others, and by 24 July 2022, 37 countries had reported 1090 probable cases to the World Health Organization (WHO), with around 57% (627 of 1090) reported in the United Kingdom and USA (273 and 354, respectively). As of August 2022, 53 children (around 5%) had required a liver transplant, and 28 (2.6%) had died.

“We could be doing more to identify causal factors to support control and prevention.”

Deirdre Kelly

Acute hepatitis is a term used to describe an acute inflammation of the liver. It can be due to a range of infectious (most commonly hepatitis viruses A to E) and non-infectious causes. Acute hepatitis is not common in young children, but it does occur and often goes unexplained.

“There have always been reports of these cases of severe unexplained hepatitis in children, but generally in low numbers,” explains Dr Philippa Easterbrook, a scientist working in WHO’s Global HIV, Hepatitis and Sexually Transmitted Infection Programme, citing studies suggesting that around 40% to 50% of all cases of severe acute hepatitis in children are of unknown cause.

In Kelly’s opinion these cases have not always attracted the attention they deserve. “As paediatricians we have tended to accept the status quo because there are so few cases and thus few unexplained cases, but each case is a tragedy for a child and their family, and we could be doing more to identify causal factors to support control and prevention,” she says.

Kelly hopes that the resources being directed at finding an explanation for the current outbreak may lead to increased investigation into other unexplained cases.

Those resources include next generation biotechnology of the kind used by teams of researchers at the University of Glasgow and University College London to elucidate possible causal factors and mechanisms involved in the current outbreak.

The aim of the studies was to look for pathogens that might be causing acute hepatitis and to establish whether there was a reason the children might be more susceptible to developing severe disease, including their genetic makeup.

One possible target in the pathogen hunt was adenovirus, which has been showing up in the blood of around half the cases with available data (363 of 760) being reported to WHO.

A common pathogen, adenovirus typically causes nothing worse than chest infections or diarrhoea in children. Why a virus which usually causes only relatively mild disease might have caused such serious problems in some children is unclear.

“Possible explanations include the children being abnormally susceptible to a virus they have not been exposed to for two years because of public health restrictions introduced as a result of COVID-19,” says Easterbrook, noting that a sudden increase in exposure to adenovirus as a result of restrictions being lifted may also have had an impact.

“A further explanation could be an abnormal or exaggerated immune response to the adenovirus leading to inflammation of the liver due to, for example, a prior SARS-CoV-2 (severe acute respiratory syndrome coronavirus 2) infection,” she adds.

The University of Glasgow team examined the biological samples taken from nine children with unexplained hepatitis (including liver samples taken from four), comparing their results with a control group of 58 children. The University College London team examined the samples taken from 28 children with unexplained hepatitis (including liver samples taken from five), comparing their results with a control group of 136 children.

The researchers came up with some intriguing results. Notable among them was the fact that all the tested samples from patients with unexplained hepatitis contained traces of an adeno-associated virus 2 (AAV2), while the control groups showed little or no trace.

Not currently known to cause disease, and provoking only a mild immune response, AAV2 is a member of the parvovirus family and requires a virus such as an adenovirus or a herpesvirus to replicate. Both adenovirus and to a lesser extent herpesvirus were found in the samples taken from the children with unexplained hepatitis, raising questions about the possible role of coinfections.

“These intriguing findings need to be evaluated now in a larger number of children,” says Easterbrook, pointing to the relatively small sizes of the two studies. “In addition, more research and data are needed to evaluate the mechanism of how AAV2 might be causing these severe cases.” Easterbrook also points out that much lower rates of adenovirus detection were reported in cases in other settings, suggesting that other mechanisms may be at work.

“We cannot allow this to fade into the background.”

Deirdre Kelly

Establishing the cause or causes of these cases is of more than academic interest. Adriana Romani, Scientific Officer Emergency Preparedness and Response, European Centre for Disease Prevention and Control (ECDC) spells it out: “Since the etiology of this disease remains largely unknown, it is not currently possible to provide specific recommendations to prevent the occurrence of further cases.”

The ECDC has been doing its part to sharpen the epidemiological picture by setting up a comprehensive surveillance system in collaboration with the WHO Regional Office for Europe. The system uses a common case definition and reporting protocol for countries in the European Region to report cases in the online European Surveillance System (TESSy). As of 28 July 2022, 508 cases of acute hepatitis of unknown etiology had been reported by 21 countries through the system, including 273 cases from the United Kingdom.

ECDC is also supporting European Union and European Economic Area Member States in developing systems to assess whether cases exceed what would normally be expected, based on the analysis of hospital data defined using the International Classification of Diseases codes to define diseases, signs and symptoms. Staff at WHO headquarters are undertaking a similar global study.

“The systems should allow for prompt detection of increased cases,” says Romani, adding that ECDC is also working with its Member States, WHO, and various clinical research networks to coordinate the collection of detailed information on cases and matched controls to test several hypotheses including the possible role of adenovirus infection.

Easterbrook applauds these efforts and would like to see more. “There is an opportunity for collaboration on similar host genetic and metagenomic studies in cases from other countries reporting higher numbers of cases,” she says, adding that work will need to move swiftly, not least because the outbreak may be on the decline, as suggested by reporting in early August.

Fewer cases will mean less data, but also less urgency to act on the part of the relevant health authorities. Already in the United Kingdom, UKHSA has stepped down from emergency to routine reporting of cases relying on reports by paediatricians.

Kelly hopes that focus can be maintained. “For those children requiring specialized attention and transplantation it's a big life event because even with the best of outcomes they will require long-term monitoring and immunosuppression. For this and other reasons we cannot allow this to fade into the background along with all the other unexplained cases.”

Dr Aymin Delgado agrees, confessing that one of the most difficult parts of her job is telling parents that their child died. “It always devastating,” she says, adding that the only comfort parents get is knowing that their child was with a team of clinicians that were doing everything possible. If they knew what they were dealing with, they’d be able to do more.

**Figure:**
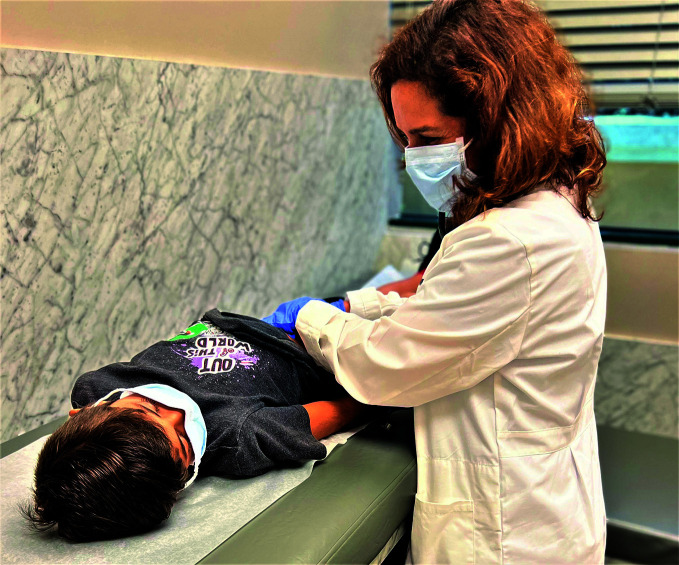
Dr Aymin Delgado examines a patient.

Courtesy Dr Aymin Delgado

